# The Treatment of Patients after Abdominal Operations

**Published:** 1895-10-12

**Authors:** Leonard A. Bidwell

**Affiliations:** Senior Assistant Surgeon to the West London Hospital, &c.


### Medical Progress and Hospital Clinics.

[*The Editor will be glad to receive offers of co-operation and contributions from members of the profession. All letters should be addressed to* The Editor, at the Office, 428, Strand, London, W.C.]

### THE TREATMENT OF PATIENTS AFTER ABDOMINAL OPERATIONS.

By Leonard A. Bidwell, F.R.C.S., Senior Assistant Surgeon to the West London Hospital, &c.

(*Continued from page* 8.)

The next point to consider is how soon should a patient be allowed to leave her bed after laparotomy. My practice is to allow her to get up at the end of a fortnight, except in cases of hysterectomy, where I usually keep her in bed for another week. In most cases the patients have left the hospital at the end of three weeks.

The wound is dressed on the tenth day and the stitches removed; long strips of strapping are then applied and worn till the end of the month. If the abdominal walls have been sutured in layers, with buried silk sutures, no abdominal belt is necessary afterwards: but after an incision more than three inches in length, which has been closed by sutures passing through the whole thickness of the walls, I usually order a belt to be worn for six or eight months. It is specially important in cases of laparotomy in the linea semilunaris that a belt should be worn if the wound has not been sutured in layers; I have known several cases of hernia where the old method of suturing was employed.

The laparotomies which I have treated on these lines consist not only of ovariotomies and hysterectomies, but also of abdominal nephrectomies, cholecystotomies, gastro-enterostomies, &c., and I consider that we do not require any special treatment for any particular region of the abdomen. With regard to hysterectomies perhaps we may have more trouble with the re-establishment of peristalsis owing to the dropping down of loops of bowel into the pelvis; however, this can be overcome by the longer use of the flatus tube, and by more frequent enemata.

I attribute the success of my operations to simplicity of treatment and to the attention paid to the re-establishment of peristaltic action by (1) early feeding; (2) the use of the flatus tube and enemata; and (3) early changes of the position of the patient in bed.

Peritonitis, the old bugbear of abdominal surgeons, ought not to occur after any simple laparotomy. Septic peritonitis can only occur if some septic matter has been introduced into the peritoneal cavity at the time of the operation, either by the operator's hands, instruments, ligatures, or sponges; except in the case of a septic tubal abscess, when it may occur after insufficient cleansing of the peritoneal cavity, prolonged drainage gives the best chance of success in these cases. The peritonitis due to the transudation of the bacillus coli commune, after damage of intestine, is best prevented by attention to the re-establishment of peristaltic action.

There is one cause of death in abdominal cases which I cannot pass over, and that is shock. This term has been somewhat loosely applied to any case which died within a few days after an operation, and so included early acute cases of septic peritonitis. In true shock the phenomena are due to the reflex dilatation of the portal system, by which nearly the whole of the blood of the body is received into the portal veins, to the detriment of the nourishment of the nerve centres. Fatal shock is in some way associated with the administration of morphia. Thus it was found by Professor Sherrington, in some experiments on division of the spinal cord in monkeys, that in all cases in which morphia was given, although the apparent shock was not so great, the animals died, while, when no morphia was given, the animals recovered from the operation.

Prevention of shock is better than cure, and its occurrence can, to a great extent, be prevented by the avoidance of exposure of the patient during the operation, by keeping the operating theatre at a temperature of 65 degrees, and by the use of a table heated by hot water. In sudden shock coming on during the operation, the best results are obtained by the infusion of from two to five pints of normal saline solution into the basilic vein, as has been recommended by Mr. Mayo Robson and others, and of the use of which I have recorded cases elsewhere. In collapse after operation, the hypodermic injection of strychnine is most efficacious. It is quite as stimulating as ether, and its effects are more lasting; moreover, it does not leave sores at the points of injection, which so frequently follow the use of ether.

A rare and quite preventible complication after laparotomy is the bursting open of the abdominal wound during convalescence. I have seen several cases in which this accident occurred when the stitches had been removed on the eighth day, and the patient had been troubled with a violent cough or vomiting. It is interesting to note that all the cases to whom this accident occurred made a perfect recovery. In one very interesting case of my own, nearly my first abdominal section, the wound, after an abdominal nephrectomy for cancer of kidney, was stitched up with rather fine boiled silk. The patient had a good deal of flatulence, and three days after the operation the nurse heard some cracking sounds, and the patient had a little abdominal pain. On examining the abdomen my house surgeon found that as three of the sutures had broken, the wound had burst, and a coil of small intestine had escaped. He promptly returned. this, and closed the wound with silkworm gut sutures. The patient made a perfect recovery, without any tendency to hernia.

Although I would not wish to imply that the care of abdominal cases should be relaxed, I maintain that there is nothing very different in such cases from any other surgical operation, and that it is not necessary to separate them from ordinaiy surgical work. In my opinion, too, an efficient surgical nurse, who is able to pass a catheter and give an enema, is quite capable of nursing any case of laparotomy without any special training.

